# Prothrombin, alone or in complex concentrates or plasma, reduces bleeding in a mouse model of blood exchange-induced coagulopathy

**DOI:** 10.1038/s41598-019-49552-9

**Published:** 2019-09-10

**Authors:** Louise J. Eltringham-Smith, Ruoying Yu, Syed M. Qadri, Yiming Wang, Varsha Bhakta, Edward L. Pryzdial, Jeffrey R. Crosby, Heyu Ni, William P. Sheffield

**Affiliations:** 1 Centre for Innovation, Canadian Blood Services, Hamilton, ON Canada; 2 Centre for Innovation, Canadian Blood Services, Toronto, ON Canada; 3 Centre for Innovation, Canadian Blood Services, Vancouver, BC Canada; 4 Department of Pathology and Molecular Medicine, McMaster University, Hamilton, ON Canada; 5 Department of Laboratory Medicine, University of Toronto and Keenan Research Centre for Biomedical Science of St. Michael’s Hospital, Toronto, ON Canada; 6 Centre for Blood Research, University of British Columbia, Vancouver, BC Canada; 7 IONIS Pharmaceuticals, Carlsbad, CA United States; 8 Present Address: Faculty of Health Sciences, Ontario Tech University, Oshawa, ON Canada

**Keywords:** Trauma, Blood proteins

## Abstract

Prothrombin complex concentrates (PCC) are fractionated plasma protein drugs that reverse warfarin anticoagulation. PCC may control more general bleeding. We sought to identify the dominant procoagulant factor in PCC *in vivo*. We tested PCC or coagulation factor (F) treatment in CD1 mice made coagulopathic by exchange of whole blood for washed red cells. Anesthetized mice were transfused with murine fresh-frozen plasma (mFFP), PCC, mixtures of human vitamin K-dependent proteins (VKDP) (prothrombin, FVII, FIX, or FX), or purified single human VKDP, immediately prior to tail transection (TT), liver laceration (LL), or intravascular laser injury (ILI). Plasma donor mice were treated with vehicle or control antisense oligonucleotide (ASO-CON) or ASO specific for prothrombin (FII) (ASO-FII) to yield mFFP or ASO-CON mFFP or ASO-FII mFFP. Blood losses were determined spectrophotometrically (TT) or gravimetrically (LL). Thrombus formation was quantified by intravital microscopy of laser-injured arterioles. PCC or four factor- (4F-) VKDP or prothrombin significantly reduced bleeding from TT or LL. Omission of prothrombin from 4F-VKDP significantly reduced its ability to limit bleeding. Mice transfused with ASO-FII mFFP demonstrated inferior haemostasis versus those transfused with ASO-FII following TT, LL, or ILI. Prothrombin is the dominant procoagulant component of PCC and could limit bleeding in trauma.

## Introduction

The restoration of haemostasis in the bleeding patient is challenging in coagulopathy, whether it arises from quantitative or qualitative deficiencies of clotting factors^1^. Since plasma contains all the soluble clotting factors, plasma transfusion seems a logical intervention in coagulopathy with bleeding, if necessary combined with platelet transfusion for concomitant thrombocytopenia and/or red cell transfusion for concomitant anemia. However, systematic reviews of randomized clinical trials (RCT) of plasma transfusion have shown little or no evidence of significant benefit^2^, and recent RCTs of prehospital plasma transfusion to trauma patients have yielded conflicting results^3,4^. Fluid volume considerations and lengthy times of infusion further limit plasma transfusion^5^. Concentrated sources of one or more clotting factors could theoretically overcome some of these limitations if those factors most needed in pan-factor deficiency could be identified.

While no single concentrated source of all soluble coagulation factors exists, a concentrate containing multiple coagulation factors is in clinical use. Prothrombin complex concentrates (PCC) are manufactured from pooled donor plasma and are enriched in the vitamin K-dependent plasma proteins (VKDP, including coagulation factors II, VII, IX, and X and anticoagulant proteins C and S)^6^. PCC infusion has replaced plasma transfusion as the recommended first-line treatment for rapid vitamin K antagonist (e.g. warfarin, phenprocoumon) reversal in many national and professional practice guidelines^7–9^, supported by RCT evidence^10,11^. Limited clinical evidence also suggests that PCC and/or fibrinogen concentrate administration may improve outcomes and reduce platelet and red cell transfusion requirements in patients with major blunt trauma^12^. Preclinical data is available from complex animal models of dilutional coagulopathy in which blood is replaced with colloid solutions, prior to traumatic and/or surgical injury. Outcomes vary by species and by the site of hemorrhagic injury. Studies in hemodiluted pigs have shown that PCC administration reduced blood loss after liver^13,14^ or bone^15^, but not spleen^15^ injury, compared to placebo. In hemodiluted rabbit models, PCC reduced blood loss from the injured kidney to a greater extent than saline or recombinant factor VIIa^16^, while neither PCC nor fibrinogen concentrates reduced blood losses from the injured liver^17^.

Previously we established a reductionist coagulopathic mouse model (Blood Exchange-induced Coagulopathy Approach, BECA) in which bleeding responds to plasma transfusion^18^. BECA mice exhibit a 5-fold reduction in all plasma proteins, along with 2- and 3- fold reductions in hematocrit and platelet counts, while maintaining a normal blood volume. Unlike other models, the BECA model isolates coagulopathy and bleeding diathesis from hemorrhagic shock and focuses on clotting factor replenishment independently of remedying hypovolemia. Transfusion of plasma from wild-type or FVIII-deficient, but not fibrinogen-deficient mice, reduced blood loss in this model^18^. Given that most coagulation factor knockout mice are not viable^19^, in the current study we explored PCC as an alternative way of determining which coagulation factors are most important in coagulopathy. We hypothesized that administering PCC or one or more components of PCC would drive thrombin generation and fibrin formation *in vivo*, restoring haemostatic control and reducing bleeding. We tested this hypothesis in BECA mice subjected to tail transection, liver laceration, or intravascular laser injury. Our results suggest that prothrombin is the most important component of PCC formulations with respect to anti-hemorrhagic efficacy *in vivo* in mice, since purified prothrombin itself replicates the anti-hemorrhagic effects of PCC and antisense oligonucleotide-mediated depletion of prothrombin in donor mouse plasma eliminates its anti-hemorrhagic efficacy.

## Results

### General approach

**Figure 1:**
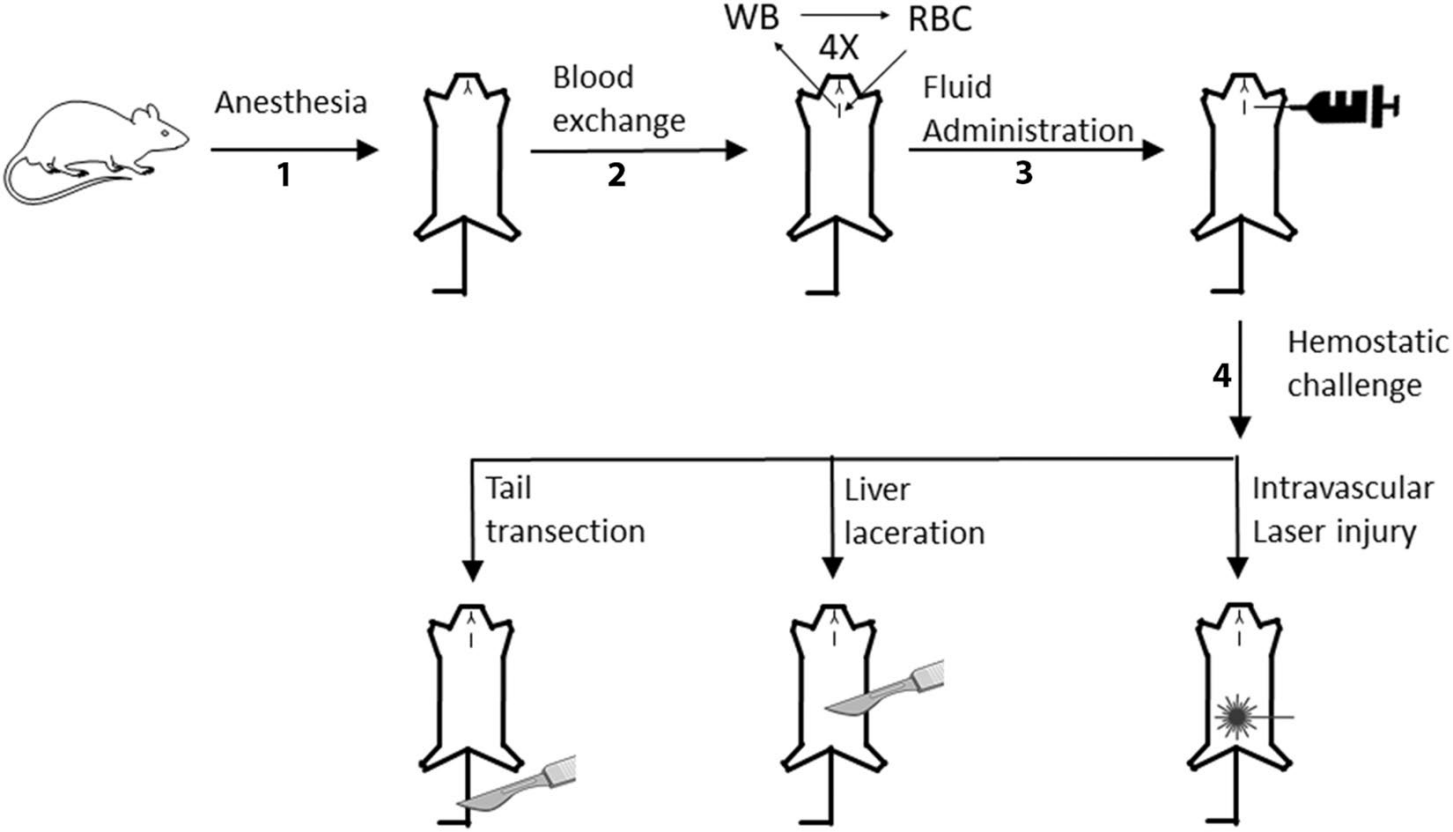
Schematic diagram of the Blood Exchange-Induced Coagulopathy Approach (BECA^18^) used in this study. Mice were: anesthetized (upright to supine, step 1); subjected to blood exchange comprising 4 × 0.5 ml exchanges of withdrawn whole blood for washed red blood cells (RBC) to induce coagulopathy (step 2); treated with 12 ml/kg of fluids (saline, plasma, PCC, VKDP, purified FIX or prothrombin) (step 3); and challenged haemostatically by either tail transection (TT), liver laceration (LL), or intravascular laser injury (ILI) of the arteriolar microcirculation of the cremaster muscle (step 4). The degree of haemostatic control was determined by either quantifying blood loss in TT and LL or measuring intravascular thrombus formation over time in ILI.

BECA is a simplified model of coagulopathy that does not involve hypovolemia or shock, but which induces a bleeding diathesis that responds to plasma transfusion. We first investigated PCC as a plasma substitute in this model, challenging treated mice with one of three haemostatic challenges: tail transection; liver laceration; or intravascular laser injury. The general approach is depicted schematically in Fig. 1.

### Comparison of mFFP and PCC in haemostatic challenges

**Figure 2:**
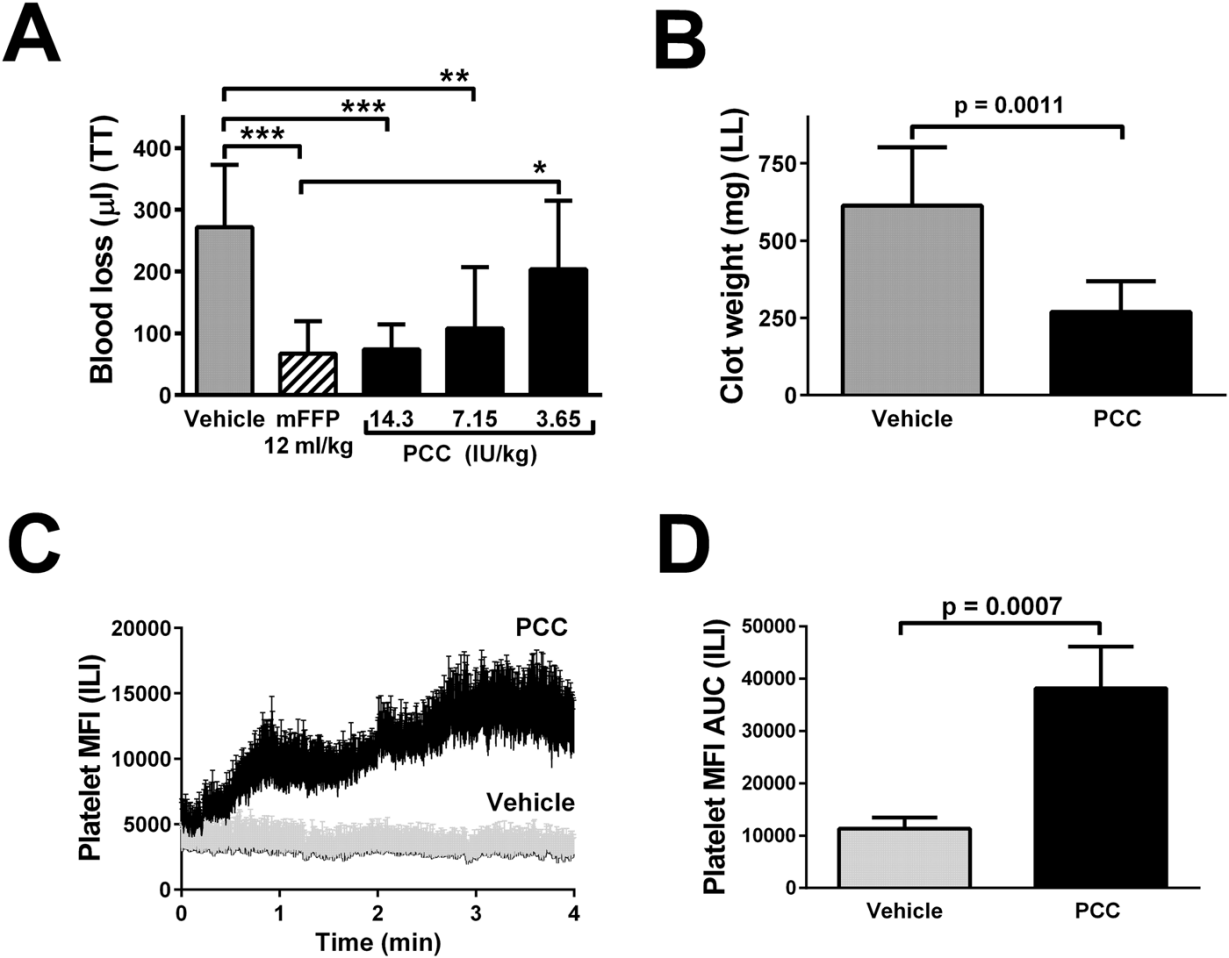
Quantification of haemostatic control following BECA and challenges and treatment with PCC. Panel A, blood loss in µl following tail transection (TT) and treatment with 12 ml/kg body weight of vehicle (Vehicle, 5% Human Albumin Solution [HAS], grey bar), mouse plasma (mFFP, hatched bar) or PCC (black bars) containing the dose in IU/kg specified below the bars. Values are presented as mean ± SD, n = 15 mice per group; *p < 0.05, **p < 0.01, ***p < 0.001 versus groups linked by horizontal capped lines by ANOVA with post-tests. Panel B, blood loss as clot weight in mg following liver laceration (LL) following treatment with 12 ml/kg vehicle (Vehicle, 5% HAS, grey bar) or 14.3 IU/kg PCC (black bar). Values are presented as in A but for n = 7. Panel C shows kinetic curves of platelet mean fluorescent intensity (MFI) detected by fluorescent intravital microscopy and measured every 15 seconds following laser injury of cremaster muscle arterioles, for receiving 14.3 IU/kg PCC or Vehicle (as in **A**,**B**). Values are presented as in (**B**) but for n = 5 or 6 thrombi; only the upper error bar (SD) is shown for clarity. In Panel (D), the curves shown in Panel (C) were quantified as the area under the platelet MFI versus time curve (AUC).

We first compared transfusion of murine plasma (mFFP) to administration of 14.3 IU/kg of PCC, a dose employed clinically for warfarin reversal^20^ (i.e. 1000 IU per 70 kg weight) in BECA mice. Figure 2A shows that this dose significantly reduced blood losses following tail transection and did not differ significantly from the reduced blood losses elicited by mFFP treatment (PCC: 73 ± 40 µl; mFFP: 67 ± 50 µl; vehicle 270 ± 100 µl, mean ± SD, n = 15). The effect persisted when the dose was reduced two-fold to 7.15 IU/kg but dissipated on four-fold dose reduction to 3.65 IU/kg.

PCC treatment (14.3 IU/kg) was also associated with a reduction in blood loss in BECA mice challenged haemostatically by liver laceration. As shown in Fig. 2B, PCC treatment led to a significant, 2.3-fold reduction in blood losses versus vehicle.

BECA mice infused with HAS vehicle showed minimal ability to respond to intravascular pulsed laser injury of the arteriolar wall of the cremaster muscle (Fig. 2C). In contrast, infusion of PCC led to an increased recruitment over time of platelets into vessel wall thrombi visualized by intravital microscopy. Quantification of thrombus size as the area under the curve of the platelet mean fluorescence intensity versus time plot revealed a significant 4.0-fold reduction in PCC-treated versus vehicle-treated mice (Fig. 2D).

### Assembly and characterization of VKDP mixtures

PCC is a mixture of plasma proteins that is a product of plasma fractionation containing not only procoagulant coagulation factors II, VII, IX, and X, but also anticoagulant proteins C and S, and other proteins^21^. To probe the minimum composition of PCC responsible for its anti-hemorrhagic effects, we required purified human coagulation factors, uncontaminated with each other. Coomassie Blue-stained non-reduced SDS-polyacrylamide gels showed the four proteins as single bands (Supplemental Fig. 1A) immunoreactive with factor-specific antibodies, with a limit of detection of 16–31 ng on immunoblots (Supplemental Fig. 1B–E). No contaminating coagulation factors were detected when 1000 ng of each preparation was probed with antibodies specific for the other three factors. These results validated the manufacturer’s claim of >95% purity and enabled the informative testing of 4-factor combinations of these vitamin K-dependent proteins (4F-VKDP). We mixed the four factors based on their labelled units of factor activity, then corrected the activity of the mixture prior to *in vivo* use by FIX assay.

### 4F-VKDP versus 3F-VKDP mixtures in haemostatic challenges

**Figure 3:**
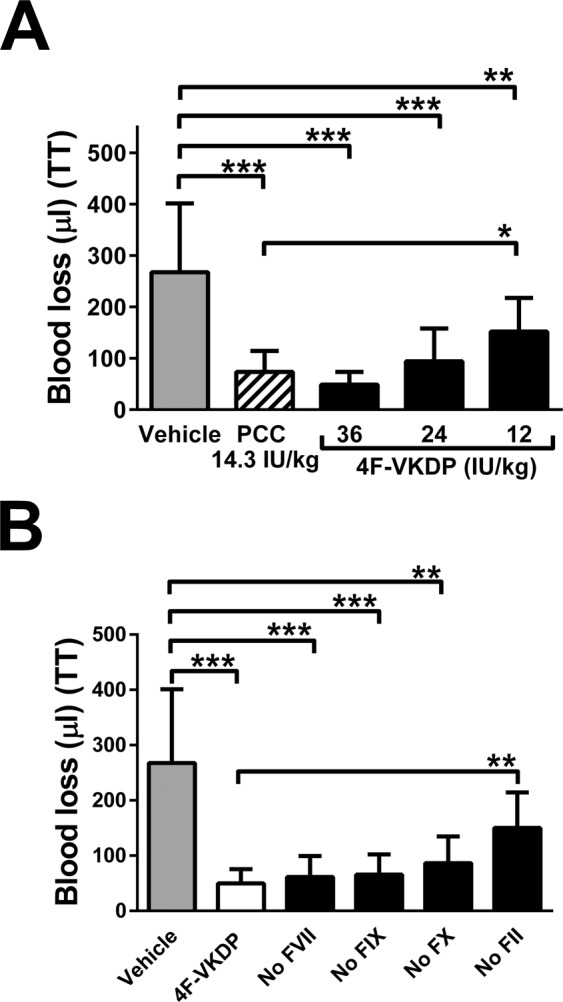
Blood losses following tail transection (TT) and treatment with 4F-VKDP or 3F-VKDP. Panel (A), blood loss in µl following tail transection (TT) and treatment with 12 ml/kg body weight of vehicle (Vehicle, grey bar), PCC (hatched bar) or 4F-VKDP (black bars) containing the dose in IU/kg specified below the bars. Values are presented as mean ± SD, n = 15 mice per group; *p < 0.05, **p < 0.01, ***p < 0.001 versus groups linked by horizontal capped lines by ANOVA with post-tests. Panel (B), as in Panel A, except that mice were treated with vehicle or 36 IU/kg 4F-VKDP (white bar) or 36 IU/kg 3F-VKDP identified by the factor they lacked (black bars, e.g. “No FVII” contained prothrombin, FIX, and FX) below the axis.

As shown in Fig. 3A, doses of 4F-VKDP of 36, 24, and 12 IU/kg all reduced blood losses significantly relative to 14.3 IU/kg PCC, but the lowest dose did not reduce it as effectively as PCC. Equivalent anti-hemorrhagic effects were therefore demonstrated between 12 and 24 IU/kg for 4F-VKDP and PCC in BECA tail transection bleeding.

Four different 3-factor (3F-VKDP) mixtures were assembled by systematically omitting one of the four vitamin K-dependent clotting factors and administered to BECA mice at 36 IU/kg to ensure maximal signal to noise ratios for comparisons. Figure 3B shows there was no significant change in the volume of blood lost for all 3F-VKDP except the 3F-VKDP (No FII) mixture, relative to 4F-VKDP. 3F-VKDP (No FII) was 3.0-fold less effective in reducing bleeding than 4F-VKDP, as evidenced by blood losses of 150 ± 60 µl versus 50 ± 30 µl (p < 0.01).

While we did not survey all the three factor combinations, we did verify that the difference between 4F-VKDP and 3F-VKDP (No FII) was also detectable in a second haemostatic challenge. BECA mice subjected to liver laceration and treated with PCC or 4F-VKDP did not differ in the amount of blood lost (270 ± 100 mg versus 340 ± 70 mg, mean ± SD, n = 7, p > 0.05) but significantly more blood was lost when 3F-VKDP (No FII) was substituted (540 ± 90 mg, p < 0.01 versus PCC and p < 0.05 versus 4F-VKDP).

### Purified prothrombin in haemostatic challenges

**Figure 4:**
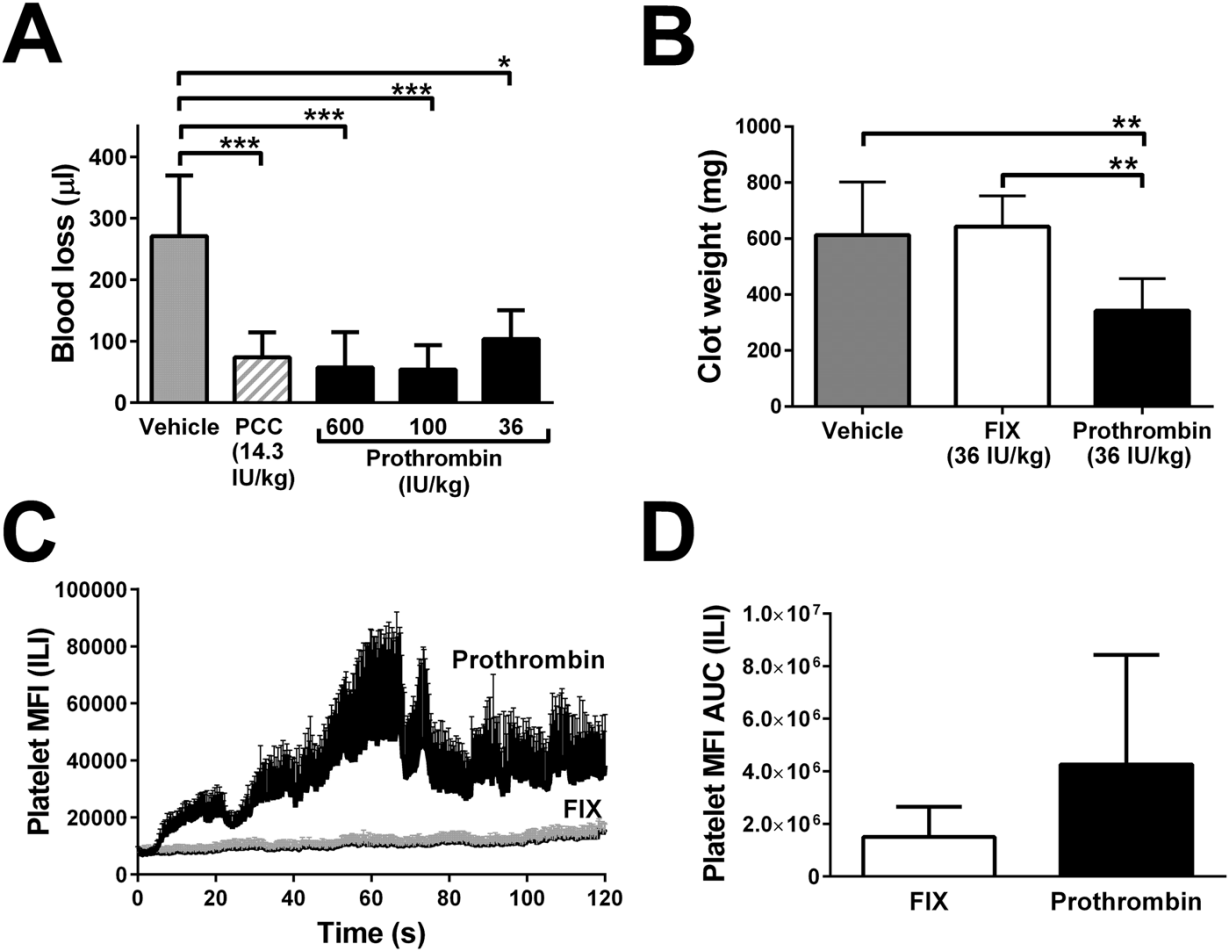
Quantification of haemostatic control following BECA and challenges and treatment with purified prothrombin. Panel (A), blood loss in µl following tail transection (TT) and treatment with 12 ml/kg body weight of vehicle (Vehicle, 5% HAS, grey bar), PCC (hatched bar), or purified human prothrombin (black bars) containing the dose in IU/kg specified below the axis. Values are presented as mean ± SD, n = 15 mice per group; *p < 0.05, **p < 0.01, ***p < 0.001 versus groups linked by horizontal capped lines by ANOVA with post-tests. Panel (B), blood loss as clot weight in mg following liver laceration (LL) following treatment with 12 ml/kg vehicle (Vehicle, 5% HAS, grey bar) or 36 IU/kg FIX (white bar) or 36 IU/kg purified human prothrombin (black bar). Values are presented as in A but for n = 7. Panel (C) shows kinetic curves of platelet mean fluorescent intensity (MFI) detected by fluorescent intravital microscopy and measured every 15 seconds following laser injury of cremaster muscle arterioles, for mice receiving 36 IU/kg PCC or FIX. Values are presented as in B but for n = 5 or 6 thrombi; only the upper error bar (SD) is shown for clarity. In Panel (D), the curves shown in Panel (C) were quantified as the area under the platelet MFI versus time curve (AUC).

Our finding that prothrombin was required for full anti-hemorrhagic effects of 4F-VKDP mixtures prompted us to ask if prothrombin alone could substitute for PCC (and therefore FFP). As shown in Fig. 4A, initially we tested a prothrombin dose of 600 IU/kg because it was employed in a published study of prothrombin treatment of hemophilia A and B mice^22^. The effects of this dose of purified prothrombin did not differ from 14.3 IU/kg PCC with respect to reducing blood loss in tail transection in BECA mice; neither did 100 IU/kg or 36 IU/kg prothrombin, although mean blood loss was slightly higher in the 36 IU/kg prothrombin-treated cohort than in the 100 IU/kg-treated cohort.

We next compared purified prothrombin to purified FIX administration in BECA mice subjected to liver laceration. As shown in Fig. 4B, 36 IU/kg FIX had no effect on blood loss following this haemostatic challenge (compare FIX to Vehicle); in contrast 36 IU/kg prothrombin significantly reduced blood loss by a factor of 1.8-fold (p < 0.01).

With respect to clot formation in the intravascular laser injury haemostatic challenge, as shown in Fig. 4C, platelet mean fluorescence plots were visibly higher in BECA mice treated with prothrombin than with FIX. When quantified as the area under the curve, this was reflected in a 2.8-fold greater thrombus profile for prothrombin- than FIX-treated mice, but the difference did not reach statistical significance.

### Generation of prothrombin-depleted plasma

The ability of purified prothrombin to reduce blood losses in coagulopathic BECA mice suggested that it could be a critical component of anti-hemorrhagically effective plasma transfusion. We sought to immunodeplete mFFP of prothrombin to test this inference using either immunoaffinity columns comprised of anti-prothrombin antibodies capable of immunodepleting human plasma of prothrombin, or immobilized DNA aptamers shown to bind human prothrombin with high affinity. Neither approach was successful (data not shown). Accordingly, we sought to deplete murine FFP of prothrombin *in vivo* by ASO treatment. As shown in Supplemental Fig. 2, plasma from untreated mice (No Tx) or those treated with a negative control oligonucleotide (ASO-CON) did not differ in plasma prothrombin levels (120 ± 30 µg/ml versus 140 ± 40 µg/ml, p > 0.05). In contrast, plasma from mice treated with ASO-FII contained 28 ± 9 µg/ml prothrombin (mean ± SD, n = 18, p < 0.001 versus ASO-CON). We therefore pooled the plasma from ASO-CON and ASO-FII donor mice for use in BECA/haemostatic challenge experiments, fixing the five-fold difference in plasma prothrombin levels between the two kinds of murine FFP.

### Prothrombin-depleted plasma transfusion and haemostatic challenges

**Figure 5:**
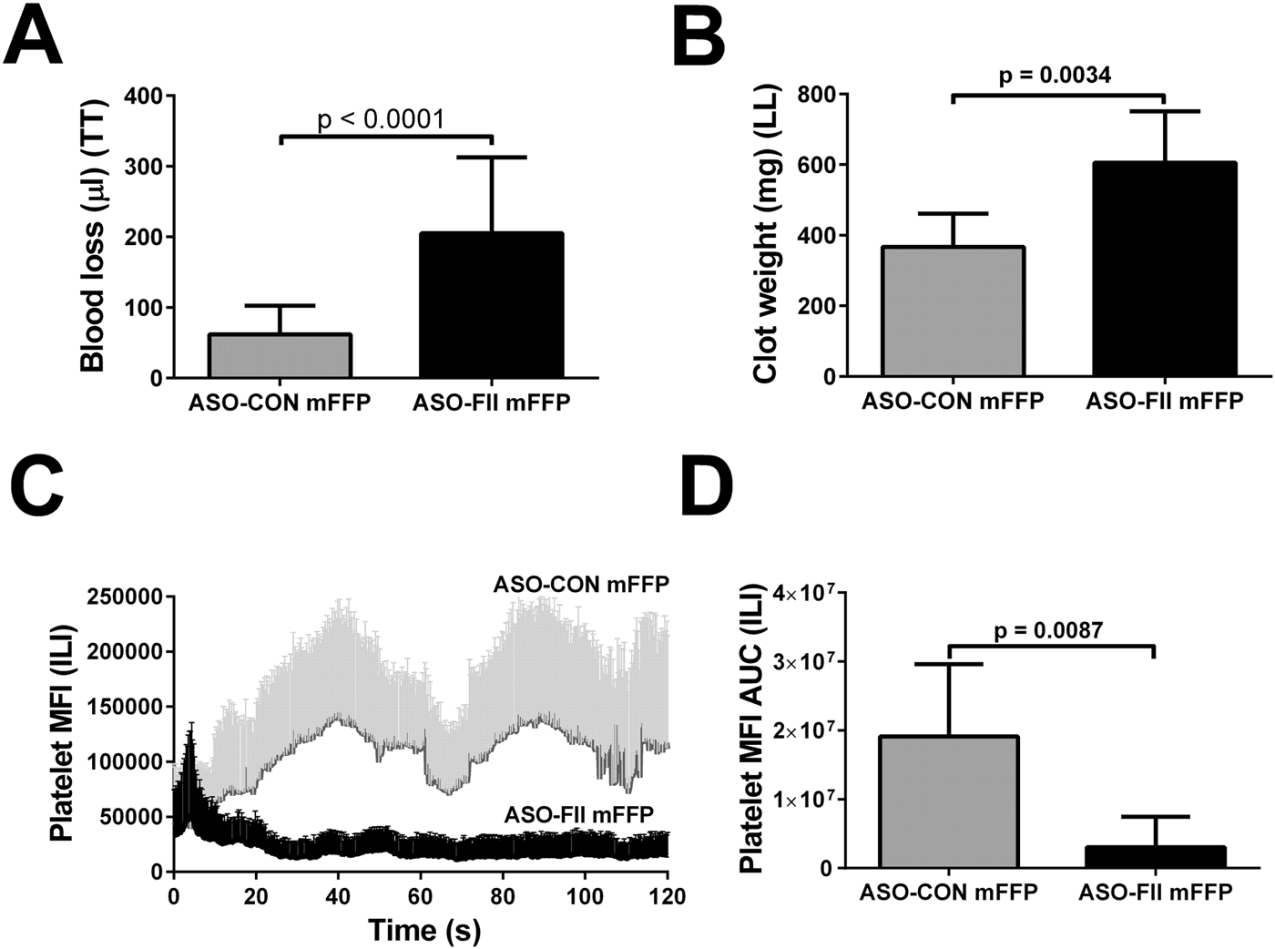
Quantification of haemostatic control following BECA with challenges and treatment with murine plasma. Panel (A), blood loss in µl following tail transection (TT) and treatment with 12 ml/kg body weight of plasma from donor mice treated with ASO-CON (ASO-CON mFFP, grey bar) or ASO-FII (ASO-FII mFFP, black bar). Values are presented as mean ± SD, n = 15 mice per group; *p < 0.05, **p < 0.01, ***p < 0.001 versus groups linked by horizontal capped lines by Welch-corrected t test. Panel (B), blood loss as clot weight in mg following liver laceration (LL) following treatment with ASO-CON (grey bar) or ASO-FII (black bar) mFFP. Values are presented as in A but for n = 7. Panel (C) shows kinetic curves of platelet mean fluorescent intensity (MFI) detected by fluorescent intravital microscopy and measured every second following laser injury of cremaster muscle arterioles, for mice receiving ASO-CON (grey line) or ASO-FII (black line) mFFP. Values are presented as in B but for n = 5 or 6 thrombi; only the upper error bar (SD) is shown for clarity. In Panel (D), the curves shown in Panel (C) were quantified as the area under the platelet MFI versus time curve (AUC).

We first compared FFP from ASO-CON-treated mice to the corresponding FPP from ASO-FII-treated mice with respect to the ability to reduce bleeding in BECA mice subjected to tail transection. As shown in Fig. 5A, the prothrombin-depleted plasma was significantly less effective than control as a fluid treatment agent; blood losses were significantly greater by 3.3-fold. Similarly, as shown in Fig. 5B, mice treated with prothrombin-depleted plasma also lost significantly more blood on liver laceration than those treated with control plasma, by 1.65-fold. With respect to intravascular laser injury, thrombus formation plots between prothrombin-depleted plasma-treated mice and those treated with control plasma diverged within 20 seconds (Fig. 5C). Quantification of the results (Fig. 5D) confirmed a significant 6.3-fold greater thrombus size over time profile associated with control plasma transfusion compared to prothrombin-depleted plasma transfusion.

### Prothrombin or PCC supplementation in normal mice

Although exploiting the BECA model was our primary approach in this study, our results prompted us to wonder if PCC or prothrombin administration could reduce bleeding in mice that were not coagulopathic at the time of injury. As shown in Supplemental Fig. 3, normal mice subjected to liver laceration injury bled less than BECA mice (compare vehicle blood losses of 200 ± 80 mg versus 600 ± 200 mg, Fig. 4B and Supplemental Fig. 3) and the blood loss was not significantly reduced by 14.3 IU/kg PCC. However, increasing the dose to 28.6 or 57.2 IU/kg PCC effected a significant reduction in blood losses  (Supplemental Fig. 3A). Similarly, a dose of 36 IU/kg purified prothrombin was sufficient to reduce blood loss on liver laceration in normal mice by 1.6-fold (p = 0.013) (Supplemental Fig. 3B).

## Discussion

We employed the BECA mouse model in this study as a simplified model of coagulopathy with a bleeding diathesis, one that we had previously shown responded to plasma transfusion^18^. BECA is not a trauma model and does not expose animals to complex pathological effects such as shock or acidosis or hypothermia. With respect to hemorrhagic shock, multiple mechanisms have been proposed to account for a possible preclinical or clinical benefit of plasma transfusion, including amelioration of factor depletion, control of excess activated protein C, reduction of hyperfibrinolysis, and endothelial repair^23,24^. BECA simplifies such considerations by avoiding shock, instead serving as an *in vivo* assay for plasma procoagulant activity. Because plasma transfusion reproducibly eliminates the bleeding diathesis in BECA, lowering the blood losses to background levels, it provides a simplified experimental venue to test the equivalence of other treatment fluids to transfusable plasma. Previously we exploited BECA to determine that FVIII but not fibrinogen was dispensable in an effective anti-hemorrhagic plasma transfusion^18^. Here, we focused on PCC, and asked which components of this concentrate were critical to restore haemostasis *in vivo*.

We found PCCs to be effective procoagulant and anti-hemorrhagic agents in BECA. Firstly, we established that 14.3 IU/kg human PCC was equivalent to 12 mL/kg murine plasma in reducing blood losses from tail transection in the BECA model. This PCC dose is among those recommended to reverse warfarin anticoagulation in humans^20^. Previously, we also demonstrated that it reversed coagulopathy and blood loss in warfarinized, non-blood-exchanged mice^25^. Secondly, PCC- treated BECA mice lost significantly less blood from liver laceration injury than vehicle-treated counterparts. Thirdly, we showed a procoagulant effect of PCC in BECA mice subjected to laser injury of the arteriolar microcirculation; thrombus size was markedly increased in PCC-treated animals. A subset of coagulation proteins represented by the partially purified human vitamin K-dependent proteins present in PCC was therefore able to drive coagulation reactions forward, to generate enough fibrin to reduce bleeding or form thrombi in several different vascular beds, in combination with residual factors circulating in the coagulopathic mouse.

We then formed the working hypothesis that the procoagulant effects of PCC in murine coagulopathy must have arisen from one or more of the vitamin K-dependent procoagulant factors within PCC. PCC are products of plasma fractionation enriched for vitamin K-dependent plasma proteins by ion exchange that captures highly negatively charged γ-glutamic acid-enriched protein domains^21^. These include not only procoagulant factors II, VII, IX, and X but also anticoagulant proteins C, Z, and S and signalling ligand Gas 6^26^, as well as other co-purifying plasma proteins such as coagulation factor V^27^. We found equivalence between PCC and VKDP mixtures containing only purified factors II, VII, IX, and X in reducing blood loss in coagulopathic mice with transected tails, in that 14.3 IU/kg PCC and 12–24 IU/kg VKDP yielded the same anti-hemorrhagic effect.

Having established anti-hemorrhagic efficacy of 4F-VKDP, we could systematically delete one factor at a time in 3F-VKDP mixtures. In BECA tail transection or liver laceration bleeding, 3F-VKDP combinations were effective if they contained prothrombin. These findings prompted us to test prothrombin alone as a treatment agent. Since Hansson *et al*. found that 30 mg/kg (300 IU/kg) recombinant human prothrombin reduced tail transection bleeding in hemophilia A and hemophilia B mice to wild-type levels^22^, we assessed 36–600 IU/kg doses in induced pan-factor deficiency, finding 36 IU/kg sufficient to reduce tail transection blood losses to mFFP-treated levels. As the same level of protection from hemorrhage was provided by 36 IU/kg prothrombin as 36 IU/kg 4F-VKDP, this suggests that the prothrombin in VKDP was responsible for the entire anti-hemorrhagic effect. This finding was specific to prothrombin, as treatment with the same amount of FIX activity had no effect.

Although our model and the reductionist approach by which we deduced the importance of prothrombin are novel, other investigators have also suggested that prothrombin is the strongest of the natural procoagulants in plasma, and by extension, PCC. Using plasmas deficient in factors II, VII, IX, or X, Xi *et al*. found that prothrombinase activity was primarily governed by prothrombin levels^28^. Butenas *et al*. drew similar conclusions in a multi-component synthetic plasma system, that prothrombin was the dominant procoagulant factor influencing thrombin generation^29^. These *in vitro* findings are supported by observational studies in rare patients with genetic prothrombin deficiencies who encounter bleeding problems, even with prothrombin activity levels as high as 18.9%^30^. Similarly, mice lacking prothrombin die *in utero* or in the perinatal period^31^, while administration of an antisense oligonucleotide directed against prothrombin mRNA leads to a dose-dependent increase in blood loss from the transected tail^32^. Our current results are therefore consistent with previous studies.

That our results were not wholly dependent on our use of the BECA model is shown by our experiments with normal mice subjected to liver laceration injury. We found that blood losses could be reduced by PCC or prothrombin administration, although higher doses were required than in BECA mice subjected to tail transection to push the non-coagulopathic system towards increased fibrin generation. Liver-lacerated BECA mice bled less when treated with high doses of purified prothrombin than with the same dose of purified human FIX, supporting the results obtained with the less severe tail transection injury. These results also reinforce the concept that critical excess coagulation factors, such as prothrombin, can drive coagulation forward via mass action.

While our primary goal was to identify which procoagulant factor in PCC was dominant for anti-hemorrhagic benefit, our findings also imply that PCC, and purified prothrombin, may have utility as treatments in traumatic injury. Prothrombin monotherapy has only been attempted in murine hemophilia A and B models to date^22^, but Mitterlechner *et al*. compared the ability of binary combinations of fibrinogen and PCC or prothrombin to generate thrombin and reduce bleeding in a coagulopathic pigs^33^. Both combinations were equally effective in reducing blood losses from liver injury.

Because our experiments suggested that prothrombin was the critical component of PCC and VKDP mixtures, we hypothesized that it would also be a critical component of anti-hemorrhagically effective plasma. We used an ASO approach to reduce prothrombin levels down to 20% of normal; in the published protocol we followed, any higher or more prolonged a dosing regimen was associated with bleeding problems in the ASO-treated mice^32^. We found a reduction in prothrombin levels to approximately 20% of normal in donor plasma, the same reduction effected by blood exchange in the BECA mice^18^. Transfusion of 30% plasma volume of this depleted plasma would therefore leave prothrombin levels at 20% of normal but would be expected to raise all other factors to 0.5/1.3, or 38.4% of normal prior to volume normalization via excretion. Previously we showed that transfusion of FVIII-deficient plasma, but not fibrinogen-deficient plasma restored haemostatic control in this model. These findings suggest that some coagulation factor or combination of factors has a critical concentration between 20% and 38.4% of normal necessary for haemostatic control^18^. FVIII is not one of these critical factors, since it declines, by dilution, to 15.4% of normal on transfusion of FVIII-free plasma that restores haemostatic control^18^. Fibrinogen is not a coagulation factor per se, as it is neither an enzyme nor a co-factor, but is instead a substrate for fibrin production^34^. Transfusion of wild-type or FVIII-deficient plasma raised fibrinogen levels to 38.4% of normal^18^, which was sufficient to restore hemostasis in the presence of normal levels of prothrombin, but insufficient at 20% normal prothrombin levels, as when prothrombin-depleted plasma from ASO-treated mice was transfused. Prothrombin was even able to drive coagulation and restore hemostasis when it was the only protein component of the treatment fluid. In this instance, fibrinogen levels (and those of all other plasma proteins) fell to 15.4% of normal, while at the lowest effective dose of 36 I.U. per kg body weight, prothrombin levels were elevated to 84.2% of normal immediately following treatment fluid infusion. This level of prothrombin must have been sufficient to generate enough thrombin to produce fibrin to form durable clots and restore hemostasis, as would be expected for an enzyme-catalyzed reaction below substrate saturation (i.e. thrombin-catalyzed conversion of fibrinogen to fibrin). Our results therefore provide support in a mouse model for the finding that both prothrombin and fibrinogen levels were critical in restoring hemostasis in coagulopathic pigs^33^.

Our study was subject to some limitations. Of necessity, we administered human proteins to mice, where less than optimal compatibility might occur. This might explain the observed need for 14.3 IU/kg PCC rather than 12 ml/kg mFFP; the latter would be expected, by definition, to contain 12 IU of both the FIX on which PCC unitage is measured, by convention, and all other factors. Nevertheless, since all other PCC and VKDP proteins were human in origin, relative comparisons between these treatments were insulated from this concern. Like many investigators, we also administered treatment agents at the time of injury, rather than after injury as is always the case clinically. We also equalized treatment volumes to simplify comparisons while remaining aware that this approach eliminated two advantages of PCC administration over plasma transfusion: lesser fluid volume; and shorter time of delivery. Finally, it is possible that our findings will not apply to the most severe of injuries, since BECA mice retained ~20% of normal levels of all coagulation factors. Further reductions in residual plasma proteins could be resistant to treatments shown to be effective in our study, but we could not assess this possibility because further reductions in plasma protein levels cause early mortality in BECA^18^.

In conclusion, our findings suggest a key role for prothrombin in PCCs, in plasma, or as stand-alone haemostatic monotherapy. Our murine data joins limited data from pig and rabbit models in supporting the potential therapeutic utility of PCC or prothrombin. Further investigations are required to determine if PCC or prothrombin administration is effective in clinically relevant models of hemorrhagic shock, building upon the findings in this reductionist coagulopathic model.

## Materials and Methods

### Animals

CD1 mice (20–25 g, Charles River, St-Constant, QC, Canada) were used for *in vivo* experiments conducted in the Central Animal Facility (McMaster University, Hamilton, ON, Canada) or in the Research Vivarium of St. Michael’s Hospital (Toronto, ON, Canada). All procedures complied with Canadian Council on Animal Care guidelines and were approved by the Animal Research Ethics Board of McMaster University (Faculty of Health Sciences) or the St. Michael’s Hospital Animal Care Committee. No anesthetized mouse was permitted to regain consciousness at any time and all were euthanized at the close of the observation periods. Equal numbers of male and female mice were employed in all experiments except for intravascular laser injury, where the choice of the cremaster muscle for intravital microscopy necessitated the use of male mice.

### Proteins

Commercial PCC (Octaplex®, Octapharma, Vienna, Austria) was diluted in 5% (wt/vol) human albumin solution vehicle (HAS; Grifols/Canadian Blood Services, Clayton, NC, USA) to standardize injection volumes and provide a total protein concentration resembling that of plasma for all mixtures of factors. Purified human factor II (FII, prothrombin), factor VII (FVII), factor IX (FIX), and factor X (FX) were obtained from Enzyme Research Laboratories (South Bend, IN, USA) and diluted in vehicle to create four factor vitamin K-dependent protein (4F-VKDP) mixtures. Three factor VKDP preparations (3F-VKDP) were made by combining three of the four coagulation factors (e.g. 3F-VKDP (No FIX) contained no FIX, etc). All fluids containing VKDP were described using PCC convention (1 IU PCC = 1 IU FIX activity)^6^ except 3F-VKDP (No FIX), in which the units referred to the other three equal activities.

### Coagulation factor assays

Coagulation factor assays specific for prothrombin, factor VII, factor IX, and factor X were carried out on PCC and 4F-VKDP preparations using a STA Compact automated coagulation analyzer (Diagnostica Stago, Asnieres, France) following the manufacturer’s directions. All reagents, including specific factor-deficient plasma and STA-Neoplastine CI Plus assays were obtained from Diagnostica Stago.

### Blood exchange-induced coagulopathy approach (BECA) model

Sequential blood exchanges were employed to create coagulopathy in mice, using the previously described Blood Exchange-induced Coagulopathy Approach (BECA)^18^. Whole blood from anesthetized mice (0.5 mL) was exchanged for washed murine red blood cells in 5% HAS, four times over 15 minutes, effecting a total 2.0 mL exchange (1.5X blood volumes). Mice tails were then immersed in 37 °C double-distilled water (dd H_2_O) for one minute. Treatment fluids (0.3 mL volume in all cases) were then injected, two minutes prior to transection of the tail at a diameter of 3.0 mm. Shed blood was collected into 37 °C dd H_2_O for fifteen minutes, then quantified in a spectrophotometer using the optical density at 490 nm.

### Liver laceration bleeding model

As previously described^35,36^ a perforating incision of 5 mm length was made using a number 10 scalpel blade into the left lobe of a surgically exposed and exteriorized anesthetized mouse liver subjected to the BECA procedure. Shed and/or clotted blood was collected into a tared plastic boat for 15 minutes prior to weighing. Any clotted blood on the surface of the lobe was then gently scraped into the boat. Protein-containing or vehicle (treatment) fluids were administered two minutes prior to liver laceration. In some experiments, mice were subjected to this procedure without previous blood exchange.

### Laser-induced arteriolar thrombus formation in BECA mice

The formation of cremaster muscle arteriolar thrombi after pulsed nitrogen dye laser injury was examined in BECA mice as previously described^18,37–39^. Thrombi were detected via incorporation of mouse platelets labeled *in vivo* using a Dylight649 Mouse Platelet Labeling Reagent (0.1 µg/g mouse body weight, Emfret Analytics, Eibelstadt, Germany). After vascular injury, real-time images of thrombus formation were visualized using an Olympus BX51WI fluorescent microscope (Olympus, Richmond Hill, ON, Canada) and were quantified using Slidebook software (Intelligent Imaging Solutions, Denver, CO, USA)^18,40^.

### Depletion of prothrombin in antisense oligonucleotide-treated mice

Antisense oligonucleotides (ASOs) were used to reduce prothrombin mRNA and circulating prothrombin concentrations in mice as previously described^32^. ASO were either ASO-FII (IONIS 401025, specific for prothrombin [Factor II, FII]) or ASO-CON (IONIS 141923, a control ASO without a mammalian mRNA target) obtained under the terms of a Materials Transfer Agreement between Canadian Blood Services and IONIS Pharmaceuticals, Carlsbad, CA, USA. CD1 mice were treated with 10 mg/kg ASO-FII or ASO-CON by subcutaneous injection twice weekly for four consecutive weeks. Anesthetized mice were then sacrificed by cardiac puncture, with blood drawn into 1/9 volume of 3.8% sodium citrate and microcentrifuged (14,000 × g for ten minutes at ambient temperature) to produce fresh-frozen plasma (FFP). Murine FFP samples was tested individually for prothrombin content by prothrombin antigen immunoassay (Affinity Biologicals, Ancaster, ON, Canada). FFP from ASO-CON- and ASO-FII-treated mice was then separately pooled and aliquoted for transfusion to BECA mice (0.3 ml per mouse, i.e. 12 ml/kg) prior to haemostatic challenges (tail transection, liver laceration, or intravascular laser injury) as described above.

### Statistical analysis

A p value > 0.05 was considered insignificant in all cases. Statistical analysis was performed using InStat version 3.06 (GraphPad Software, San Diego CA) and graphs were generated using Prism 4.03 software (GraphPad Software). Data sets were assessed for normality of data distribution and similarity of standard deviations. Data sets of more than two groups passing both conditions were compared using ANOVA with Tukey post-tests, while failing data sets were compared using non-parametric Kruskal-Wallis tests with Dunn’s post-tests. Data sets comprising two data sets were compared by Welch-corrected t tests.

## Supplementary information

Supplementary Information

## Data Availability

All datasets generated during and/or analysed during the current study are available from the corresponding author on reasonable request.
