# Supplementary material for: Prothrombin, alone or in complex concentrates or plasma, reduces bleeding in a mouse model of blood exchange-induced coagulopathy
Source: Sci Rep. 2019 Sep 10;9:13029. doi: 10.1038/s41598-019-49552-9 (PMC6736877; doi:10.1038/s41598-019-49552-9)
Supplement: Supplementary file 1 — Supplementary Information [file 41598_2019_49552_MOESM1_ESM.pdf]

Supplementary Information to:

Prothrombin, alone or in complex concentrates or plasma, reduces bleeding in a mouse model of blood exchange-induced coagulopathy

Louise J. Eltringham-Smith[1,4], Ruoying Yu[2,5], Syed M. Qadri[1,4,8], Yiming Wang[2,5], Varsha Bhakta[1], Edward L. Pryzdial[3,6], Jeffrey R. Crosby[7], Heyu Ni[2,5], and William P. Sheffield[1,4,*].

[1]Centre for Innovation, Canadian Blood Services, Hamilton, ON[1], Toronto ON[2], and Vancouver BC[3], Canada,

[4]Department of Pathology and Molecular Medicine, McMaster University, Hamilton, ON, Canada,

[5]Department of Laboratory Medicine, University of Toronto and Keenan Research Centre for Biomedical Science of St. Michael's Hospital, Toronto, ON, Canada,

[6]Centre for Blood Research, University of British Columbia, Vancouver, BC, Canada, and

7IONIS Pharmaceuticals, Carlsbad, CA, United States, and

[8]Current address: Faculty of Health Sciences, Ontario Tech University, Oshawa, ON, Canada.

Supplemental Figure Legends

Supplemental Figure S1. Electrophoretic and immunological characterization of purified human coagulation factor preparations. (A) Human coagulation factors (1000 ng each, identified above the lanes) were electrophoresed without reduction on a 10% SDS-polyacrylamide gel (Gel) stained with Coomassie Brilliant Blue. Markers (M), at left, in kDa, are: 200; 150; 120; 100; 85; 70; 60; 50 (darker band); 40; and 30. (B-E) Replica gels containing the same preparations analyzed in A were electrophoresed and immunoblotted with antibodies identified below each panel. The amounts and nature of the purified proteins loaded are identified, in ng, above each lane.

Supplemental Figure S2. Prothrombin concentrations in plasma of mice treated with ASO. Prothrombin concentrations (in μg/ml) were determined by immunoassay in plasma from mice without treatment (open circles, No Tx) or treated with ASO-CON (black squares) or ASO-FI (black triangles). Each data point represents one mouse. Horizontal lines indicate the mean. ***, $p < 0.001$ by Welch-corrected t test.

Supplemental Figure S3. Blood loss as clot weight in mg following liver laceration (LL) in normal mice not subjected to BECA, following treatment with vehicle (white bar) or PCC (black bars) at the doses shown, in IU/kg, below the axis. Values are presented as mean ± SD, n=7 mice per group; **$p < 0.01$, between groups linked by horizontal capped lines by ANOVA with post-tests. Panel B, as in Panel A except normal, non-BECA mice received vehicle (white bar) or 36 IU/kg purified human prothrombin (black bar) and the p value is shown above the capped line connecting the two data sets (by Welch-corrected t test).

Supplemental Figures

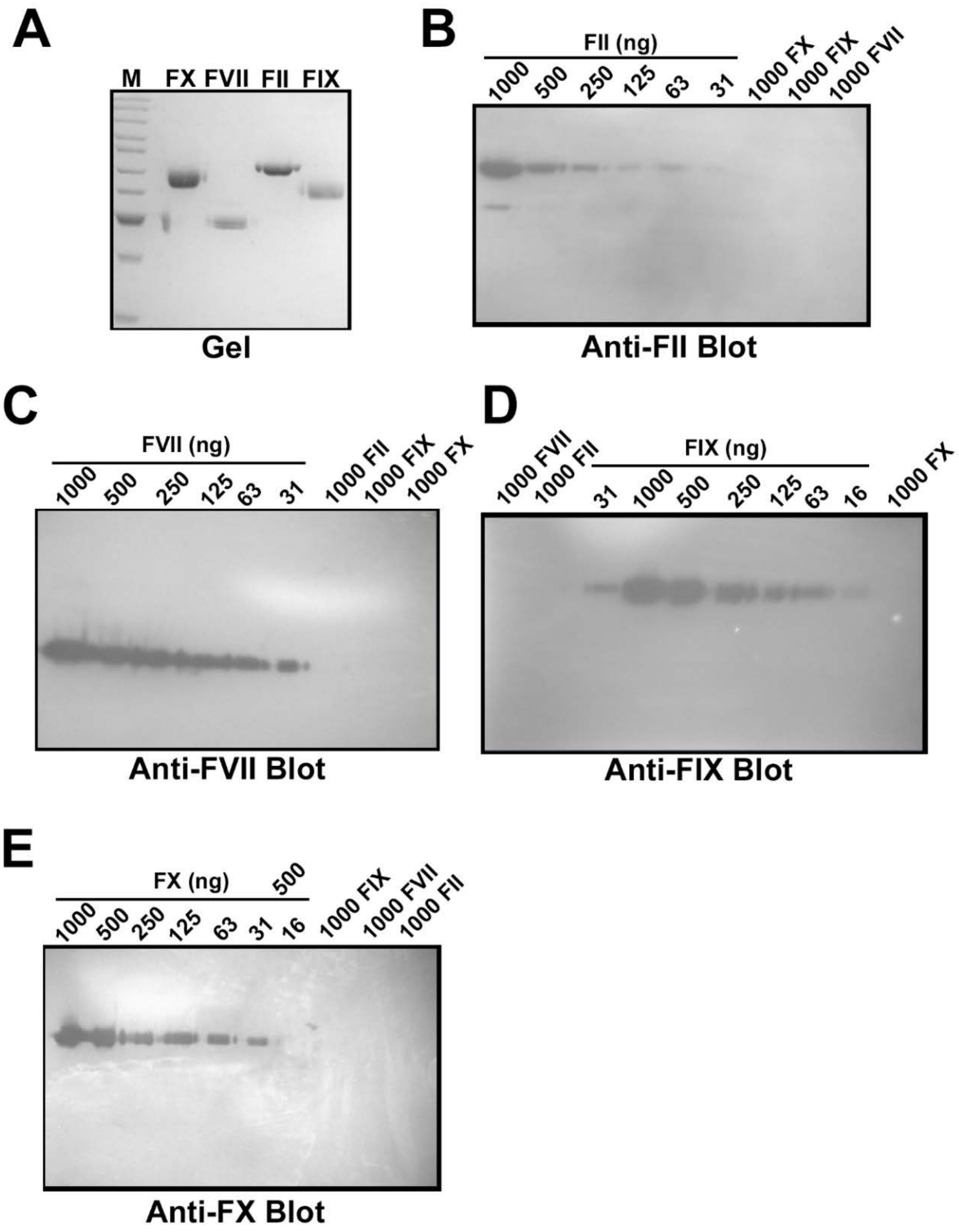

Supplemental Figure 1

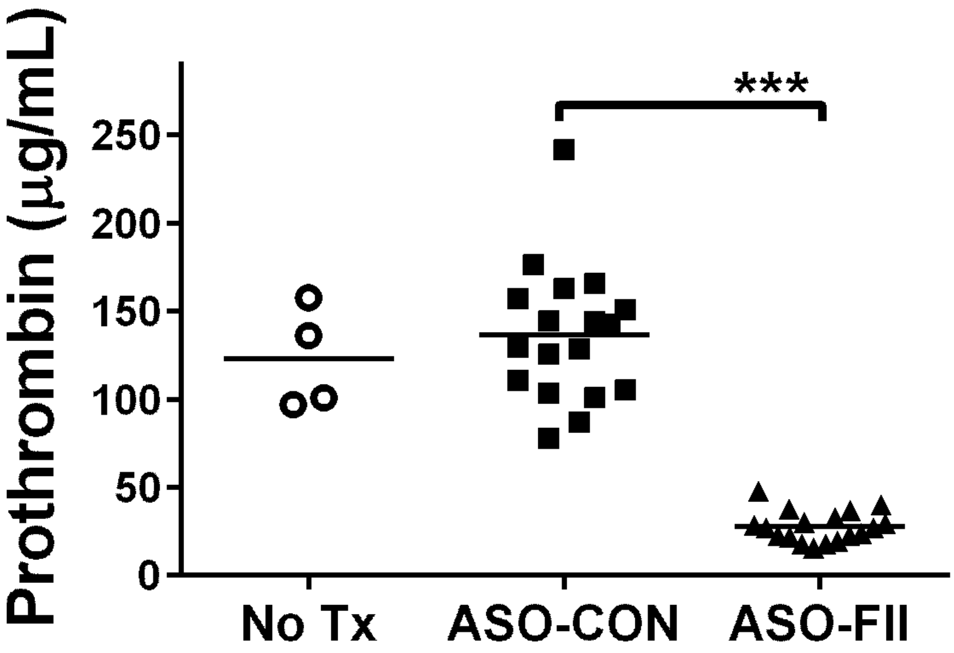

Supplemental Figure 2

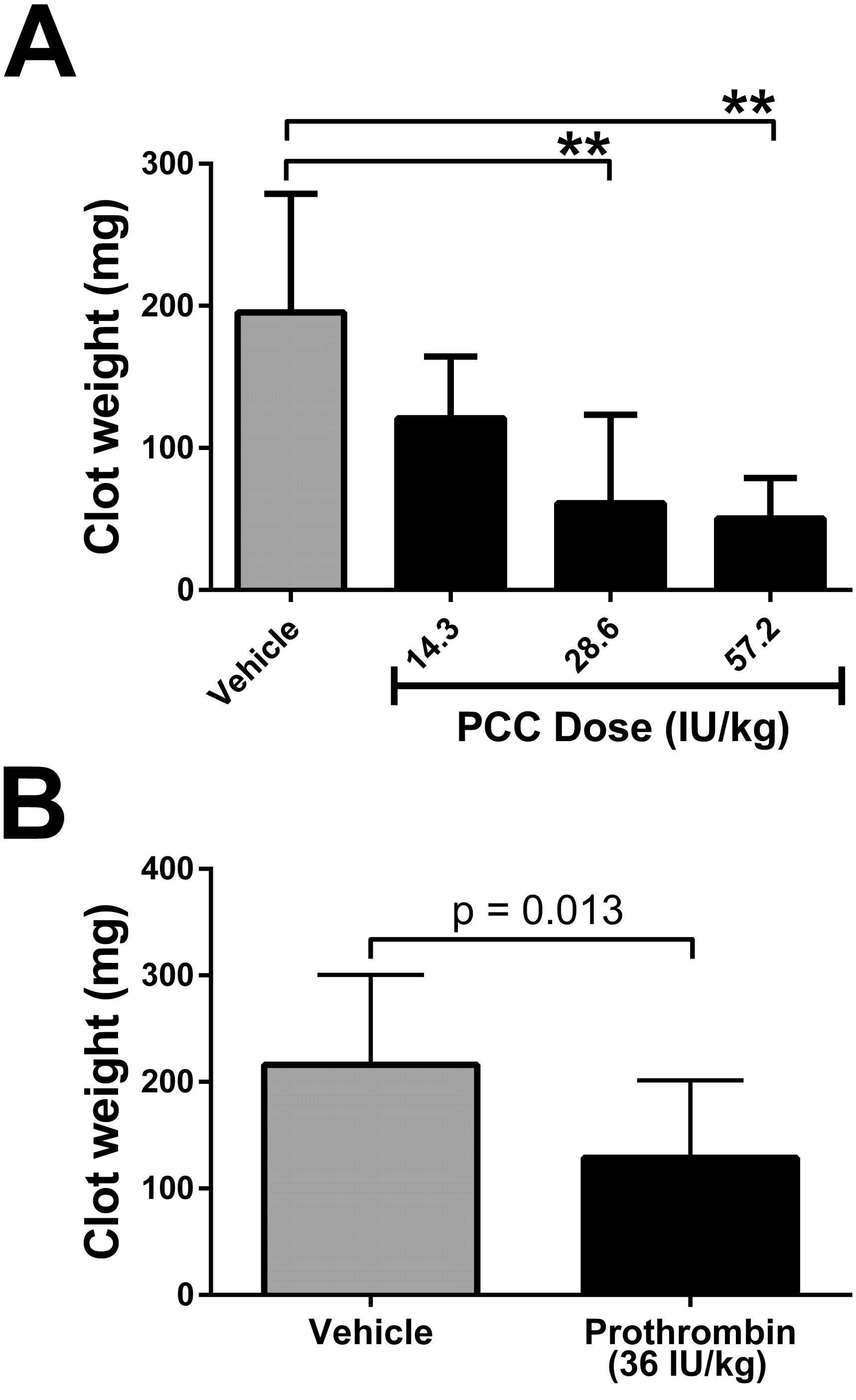

Supplemental Figure 3
